# Lack of direct association between oral mucosal lesions and SARS-CoV- 2 in a cohort of patients hospitalised with COVID-19

**DOI:** 10.1080/20002297.2022.2047491

**Published:** 2022-03-10

**Authors:** Gabriela Schwab, Michelle Palmieri, Rodrigo M. Zerbinati, Dmitry J. S. Sarmento, Thais Reis, Karem L. Ortega, Italo T. Kano, Rafael A. V. Caixeta, Bengt Hasséus, Dipak Sapkota, Roger Junges, Simone Giannecchini, André L. F. Costa, Sumatra M. C. P. Jales, José A. L. Lindoso, Camila Barros Gallo, Paulo H. Braz-Silva

**Affiliations:** a Laboratory of Virology (Lim-52-hc-fmusp), Institute of Tropical Medicine of São Paulo, School of Medicine, University of São Paulo, São Paulo, Brazil; b Department of Stomatology, School of Dentistry, University of São Paulo, São Paulo, Brazil; c School of Dentistry, State University of Paraíba, Araruna, Brazil; d Department of Oral Medicine and Pathology, Institute of Odontology, University of Gothenburg, Gothenburg, Sweden; e Institute of Oral Biology, Faculty of Dentistry, University of Oslo, Oslo, Norway; f Department of Experimental and Clinical Medicine, University of Florence, Florence, Italy; g Postgraduate Program in Dentistry, Cruzeiro Do Sul University, São Paulo, Brazil; h Division of Dentistry, Hospital Das Clínicas da Faculdade de Medicina da Universidade de São Paulo – Hcfmusp, School of Medicine, University of São Paulo, São Paulo, Brazil; i Institute of Infectious Diseases Emilio Ribas, São Paulo, Brazil; j Laboratory of Protozoology (Lim-49-hc-fmusp), Institute of Tropical Medicine of São Paulo, School of Medicine, University of São Paulo, São Paulo, Brazil; k Department of Infectious Diseases, School of Medicine, University of São Paulo, São Paulo, Brazil

**Keywords:** Oral ulcer, opportunistic infections, COVID-19, HSV-1, SARS-CoV-2

## Abstract

**Background:**

COVID-19 is a disease affecting various human organs and systems, in which the virus seeks to interact with angiotensin-converting enzyme 2 receptors. These receptors are present in the oral cavity, but the direct relationship between such an interaction and possible oral manifestations of COVID-19 is still unclear.

**Aim:**

The present study evaluated oral manifestations in a cohort of COVID-19 patients during the period of hospitalisation.

**Methods:**

In total, 154 patients presenting moderate-to-severe forms of COVID-19 had their oral mucosa examined twice a week until the final outcome, either discharge or death. The oral alterations observed in the patients were grouped into Group 1 (pre-existing conditions and opportunistic oral lesions) and Group 2 (oral mucosal changes related to hospitalization).

**Results:**

Oral lesions found in the patients of Group 1 are not suggestive of SARS-CoV-2 infection as they are mainly caused by opportunistic infections. On the other hand, oral alterations found in the patients of Group 2 were statistically (*P* < 0.001) related to intubation and longer period of hospitalisation.

**Conclusion:**

It is unlikely that ulcerative lesions in the oral cavity are a direct manifestation of SARS-CoV-2 or a marker of COVID-19 progression.

## Introduction

Months following the advance of the pandemic caused by the novel Severe Acute Respiratory Syndrome Corona Virus 2 (SARS-CoV-2), combined with the efforts of health care professionals and researchers, it was rapidly revealed that the mechanism of invasion of human cells by SARS-CoV-2 occurs through interaction with angiotensin-converting enzyme 2 receptors (ACE2) [1]. ACE2 receptors are found in several human tissues [2], which explains the extra-pulmonary extension of COVID-19 affecting other organs such as kidneys, brain, heart, gastrointestinal tract and blood vessels [3]. In the oral cavity, these receptors were also identified in tongue, periodontal tissue and salivary gland ducts [4,5].

A number of reports have been published on the possible oral manifestations of SARS-CoV-2 infection, such as intra-oral and labial aphthous-like ulcers suggestive of viral infection, petechiae and erythematous macules, blood blisters, depapillation on the tongue dorsum, reduction of the salivary flow resulting in xerostomia, and sensory disorders (e.g. dysgeusia, hyposmia and anosmia) [6–9; 10–13, 14. Dysgeusia and xerostomia are the main oral manifestations observed in COVID-19 patients [15].

The overall imbalance caused by COVID-19, either through the direct action of the virus or through the resulting damages to endothelial cells and immune response deregulation, can worsen pre-existing conditions [16,17]. In addition, patients hospitalised for a long period of time, especially those undergoing invasive mechanical ventilation without the use of oral hygiene protocols, can be subject to several types of acute oral changes such as dryness, erythema, opportunistic infections, bleeding, ulceration [18] as well as long-term sequelae such as tooth loss and periodontal disease [19].

Considering these processes, it is not known whether oral lesions observed in patients positive for SARS-CoV-2 are correlated to this viral infection. Therefore, the objective of the present study was to assess oral manifestations in a cohort of patients with COVID-19 complications throughout their hospitalisation.

## Materials and methods

### Ethical aspects

This study was performed according to the Declaration of Helsinki and approved by the Research Ethical Committee of the Emilio Ribas Institute of Infectious Diseases and School of Dentistry of the University of São Paulo according to protocol number CAAE 35589320.6.0000.0061. The volunteers were informed on the objectives, propositions and conditions of the study, and those who accepted to participate in the study signed an informed consent form. Collection of demographic and clinical data, examination of the oral cavity and saliva collection were performed after the volunteers understood the study protocol and accepted to participate.

### Recruitment of volunteers

Patients admitted to the Emilio Ribas Institute of Infectious Diseases in the city of São Paulo from January 13 to May 28 of 2021 and who were RT-PCR positive for SARS-CoV-2 by means of a nasopharyngeal swab in the past five days were invited to participate in the study. Individuals younger than 18 years of age and pregnant women were excluded.

Patients diagnosed with COVID-19 were classified depending on the severity of the disease as follows: 1) mild, characterised by presence of influenza-like symptoms, normal radiological examination and absence of dyspnoea, 2) moderate, characterised by presence of influenza symptoms associated with mild-moderate pulmonary impairment (<50%) measured with computed tomography and oxygen saturation >93% in room atmosphere, and 3) severe, characterised by respiratory frequency greater than 30 breaths per minute, oxygen saturation <93% in room atmosphere, and severe pulmonary impairment (>50%) measured with computed tomography. In the present study, only inpatients presenting the moderate and severe forms of COVID-19 were followed up.

### Saliva collection and molecular analysis of SARS-CoV-2

Specimens of saliva were obtained from the enrolled patients by using a cotton pad device – Salivette™ (Sarstedt AG & CO. KG, Nümbrecht, Germany). The patients were instructed to keep the cotton roll inside the mouth for 90 s and then place it into a tube. Next, analysis of the saliva was conducted by centrifuging the tube at 1,000 *g* for 5 s and the saliva specimens were aliquoted and stored at −80°C after centrifugation. Total RNA was extracted from 200 μL of the saliva specimen by using the PureLink™ Viral RNA/DNA Mini Kit (Invitrogen, Life Technologies Ltd., UK). Detection of SARS-CoV-2 RNA was performed by using the SARS-CoV-2 RT-qPCR Reagent kit (Perkin Elmer, Turku, Finland) according to the manufacturer’s instructions.

### Inspection of the oral cavity

Intra-oral inspection was conducted by two examiners with experience in oral medicine, who observed directly the oral mucosa (lips, jugal mucosa, tongue, buccal floor, hard and soft plate) by using indirect light (flashlight), gauze, wooden spatulas and mouth openers depending on the patient’s condition (trismus, bleeding, intubation injuries, pain, pronation). This procedure was performed in the initial appointment (inclusion phase of the study) followed by further evaluations twice a week until the final outcome (discharge or death).

The alterations found in the oral mucosa were divided into two groups: Group 1, with patients presenting pre-existing conditions and opportunistic oral lesions (e.g. pilous tongue, geographic tongue, inflammatory fibrous hyperplasia, pseudomembranous candidiasis, angular cheilitis, recurrent labial and intra-oral herpes simplex virus infections, and Group 2, with patients presenting with oral mucosal changes related to hospitalisation (dryness, erythema, atrophy, cracked mucosa, presence of loose or solid secretions, petechiae, spontaneous oral haemorrhage, blood clots and traumatic ulcers).

Additional examinations were performed whenever necessary, such as exfoliative cytology – Papanicoulau stain and Periodic Acid-Schiff (PAS) stain (Sigma-Aldrich, Inc, St. Louis, MO), and molecular test (PCR) for HSV-1 DNA detection [20], for definitive diagnosis of the lesion. Mucosal changes were photographed with a smartphone camera.

In addition to inspection of the oral mucosa, the oral condition of the patients was evaluated for presence of dental prosthesis (total or removable), orthodontic appliances and infectious odontogenic foci (e.g. caries, residual roots and abscesses).

### Statistical analyses

Considering the grouping of the patients on the basis of oral mucosal changes into Group 1 (pre-existing conditions and opportunistic oral lesions) and Group 2 (oral mucosal changes related to hospitalisation), an initial descriptive analysis was conducted for stratification of the variables.

McNemar’s test was used to assess changes in the prevalence of oral alterations during the follow-up until the final outcome in each group. Pearson’s chi-square test was used for associations between type of oxygen support and the presence of oral alterations regarding both groups of patients. Student’s t-test was used to assess the association between time of hospitalisation and presence of oral alterations in both groups. All statistical analyses were conducted by using the IBM SPSS software, version 24.0, with *P* values <0.05 being considered to be statistically significant.

## Results

The final cohort consisted of 154 patients diagnosed with COVID-19, all admitted due to complications and followed up throughout the period of hospitalisation until the final outcome (i.e. discharge or death). The mean inpatient time was 7.52 ± 12.53 days, varying from 1 to 95 days. At the first evaluation, the patients had COVID-19 symptoms for 12.77 ± 5.20 days, on average, with cough, dyspnoea and fever being the main symptoms observed (Table 1).

**Table 1.** Clinical and demographic characteristics of the participants in the study

Variable		n	%
Gender	Male	92	59.7
	Female	62	40.3
Smoking	Present	4	2.
	Never	132	85.7
	Past	18	11.7
Alcoholism	Present	14	9.0
	Never	135	87.7
	Past	5	3.3
Vaccinated aginst SARS-CoV2	Yes	15	9.7
	No	139	90.3
Type of ward	General ward	84	54.5
	ICU	70	45.5
Breathing support	Room atmosphere	23	14.9
	Oxygen support with nasal catheter	100	64.9
	Orotracheal intubation	31	20.1
Outcome	Discharge	130	84.4
	Death	24	15.6
Comorbidities	Yes	127	82.5
	No	27	17.5
Total		154	100
COMORBIDITIES:			
Systemic arterial hypertension – 48.7% (75/154)			
Obesity – 39% (60/154)			
Diabetes mellitus – 28.6% (44/154)			
Pulmonary diseases – 6.5% (10/154)			
Hypothyroidism – 6.5% (10/154)			
HIV-positivity – 4.5% (7/154)			
Dyslipidaemia – 3.9% (6/154)			
SYMPTOMS:			
Cough – 72.7% (132/154)			
Dyspnoea – 63.0 (97/154)			
Fever – 53.9% (83/154)			
Anosmia – 14.3% (22/154)			
Ageusia – 11.0% (17/154)			

The majority of the patients were male (59.7%) with a mean age of 54.60 ± 13.93 years old, varying from 20 to 88 years. Only 15 (9.7%) participants had been vaccinated against SARS-CoV2, in which 11 received the first dose (i.e. SINOVAC or AstraZeneca). The distribution of patients regarding the level of provided care was similar, being 54.5% in the general ward and 45.5% in the intensive care unit (ICU). Patients needing oxygen support represented the majority of the samples, with 64.9% on nasal catheter and 20.1% undergoing orotracheal intubation. The majority of the patients had at least one comorbidity (82.5%), in which systemic arterial hypertension, obesity and diabetes mellitus were the most frequently observed. All these data are shown in Table 1.

RT-PCR analysis of the saliva specimens showed that 67.5% (104/154) of the patients were positive for SARS-CoV-2, revealing the presence of the virus in the oral cavity at the inclusion phase of the study. Of the 104 positive cases, 49 (47.1%) were in-patients in general wards and 55 (52.9%) were patients in ICU.

Evaluation of the oral conditions showed that 20.8% of the patients wore dental prosthesis, 3.9% wore orthodontic appliances and 15.6% had some focus of odontogenic infection in the oral cavity (Table 2).

**Table 2.** Oral clinical characteristics of the participants in the study at the inclusion phase

Variable		N	**%**
Dental prosthesis	Yes	32	20.8
	No	122	79.2
Orthodontic appliance	Yes	6	3.9
	No	148	96.1
Dental infection focus	Yes	24	15.6
	No	130	84.4
Total		154	100

**Figure 1.**
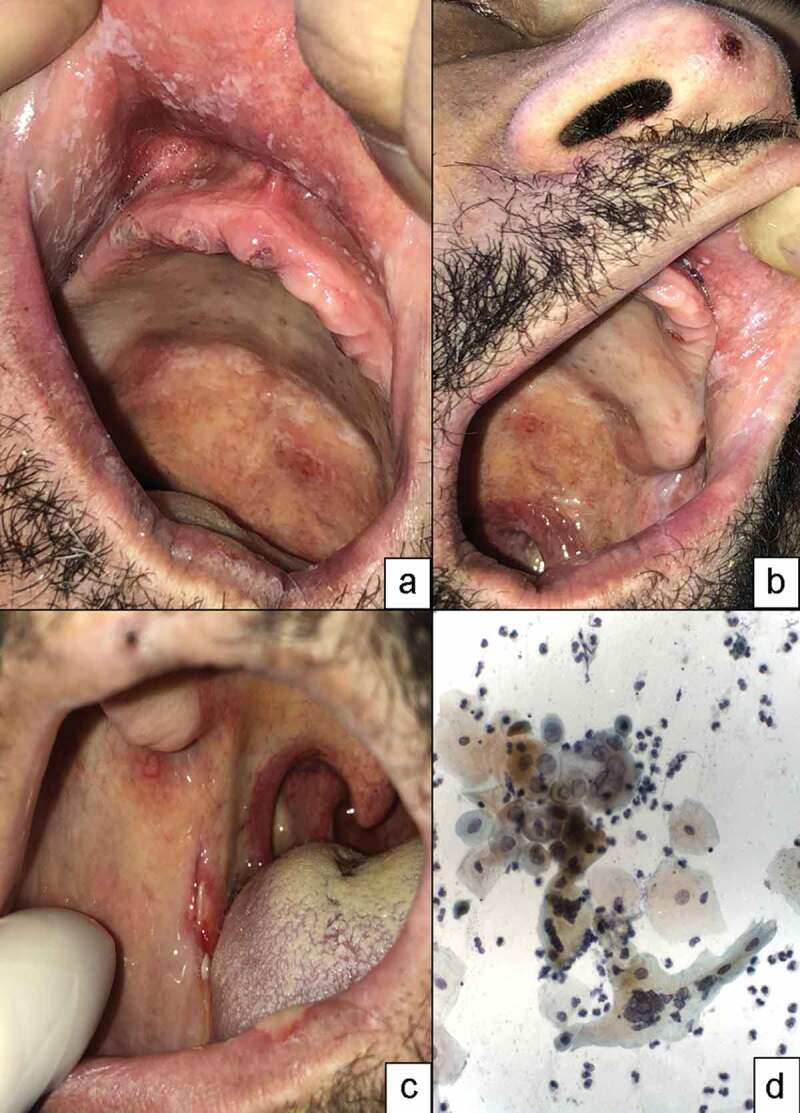
Oral lesions in COVID-19 patients hospitalised in ICU. male 62-year-old patient positive for HIV (risk of comorbidity for COVID-19) presented fever, cough, headache, dyspnoea, dysgeusia and myalgia as initial symptoms of COVID-19, remaining hospitalised in ICU for 25 days until the final outcome (i.e. discharge). A: at the first evaluation of the oral cavity, it was observed that white plaques were scattered all over the mucosa, being removed by scraping. clinical diagnosis indicated pseudomembranous candidiasis and systemic antifungal treatment was performed (single dose of fluconazole 150 mg). B: at the fourth evaluation (after 7 days), pseudomembranous candidiasis was found to be recurrent and vesiculobullous lesions were observed intra-orally in the hard and soft palate and extra-orally at the nasal apex as a crust. clinical diagnosis indicated recurrent herpes simplex and the lesions were swabbed for cytological evaluation. C: At the fifth evaluation (after another 2 days), the lesions looked like multiple and superficial ulcers on the labial and jugal mucosa, covered with fibrinopurulent exudate and surrounded by an erythematous halo, being very symptomatic. despite being hospitalised in ICU, the patient was undergoing oxygen supportive therapy and was responsive. the count of CD4 + T lymphocytes dropped from 460 to 64. D: Exfoliative cytology had a cytopathic effect compatible with HSV-1 infection showing syncytial multinucleated giant cells with a ‘ballooning’ cytoplasm (Papanicolaou x 400). the cytopathological diagnosis of HSV-1 infection was confirmed by PCR (polymerase chain reaction). the patient started treatment with fluconazole 150 mg/week and valacyclovir 500 mg, resulting in regression of the lesions within 10 days after the beginning of the therapy.

With regard to oral mucosal alterations, Group 1 had three (1.9%) patients with pre-existing conditions such as pilous tongue, geographic tongue and inflammatory fibrous hyperplasia, all clinically diagnosed. Seven (4.5%) patients had opportunistic oral infections such as pseudomembranous candidiasis and herpes simplex, which were confirmed by means of exfoliative cytology and PCR for HSV-1 during hospitalisation (Figure 1). The definitive diagnosis of recurrent intra-oral herpes was not possible in only one patient, who had been discharged from the hospital before saliva collection. In Group 2, four (2.6%) patients had oral mucosal changes related to hospitalisation throughout the period of follow-up.

Table 3 shows the patients and their oral mucosal alterations observed during the period of follow-up. Notably, the alterations found in Group 1 are not suggestive of any association with infection by SARS-CoV-2.

**Table 3.** Characteristics of the patients and respective oral mucosal alterations observed in the cohort of hospitalised COVID-19 patients

Ranking	Gender	Age	Comorbidity	Vaccine	Hospitalisation	Oral infection focus	Hospital days/outcome	Number of evaluations	Initial evaluation of the lesion	Follow-up of the lesion
Group 1	M	57	Yes	No	General ward O_2_ support	No	4 Discharge	2	Recurrent labial herpes	Absent
	F	69	Yes	No	General ward O_2_ support	No	1 Discharge	1	Recurrent labial herpes	N/E
	F	59	Yes	No	General ward O_2_ support	No	7 Discharge	2	Absent	Candidiasis
	F	51	Yes	No	General ward O_2_ support	No	1 Discharge	1	Pilous tongue	N/E
	F	69	Yes	No	ICU O_2_ support	No	4 Discharge	2	Absent	Ulcer on the tongue dorsum (recurrent intraoral herpes at the 2^nd^ evaluation).
	M	62	Yes	No	ICU O_2_ support	No	25 Discharge	6	Pseudomembranous candidiasis	Pseudomembranous candidiasis (persisted until the 2^nd^ evaluation, but with recurrence at the 4^th^ evaluation) and recurrent intraoral herpes (at the 4^th^ evaluation, persisting until the 6^th^ evaluation)
	F	38	Yes	No	General ward O_2_ support	No	4 Discharge	2	Recurrent labial herpes	Absent
	F	44	Yes	No	General ward O_2_ support	No	4 Discharge	2	Candidiasis (angular cheilitis)	Absent
	M	58	Yes	No	ICU O_2_ support	No	34 Discharge	8	Recurrent labial herpes	Recurrent intraoral herpes (persisted until the 3^rd^ evaluation)
	M	50	Yes	Dose 1	ICU O_2_ support	Yes	8 Discharge	3	Geographic tongue	Geographic tongue
	F	68	Yes	Dose 1	ICU O_2_ support	No	1 Discharge	1	IFH	IFH
Group 2	F	65	Yes	No	ICU Intubation	No	95 Death	20	No Dry oral mucosa with bleeding and trismus	Traumatic ulcer (6^th^ evaluation)
	M	76	Yes	No	ICU Intubation	No	1 Death	1	Traumatic ulcer, oral bleeding, erosion at the bottom of the sulcus on the left side, bleeding crusts, dry lips	N/E
	M	38	No	No	ICU Intubation	Yes	1 Death	1	Traumatic ulcer, ulcerative lesion in the lower labial mucosa (central region) due to trauma from the orotracheal tube, dry lips	N/E
	M	34	Yes	No	ICU Intubation	No	60 Discharge	13	Traumatic ulcer in the lower lip, clots and bleeding crusts in the lips, dry lips	Traumatic ulcer (persisted until the 2^nd^ evaluation)
	M	35	No	No	ICU Intubation	No	7 Discharge	3	Absent	Traumatic ulcer (at the 2^nd^ and 3^rd^ evaluations)

IFH: Inflammatory fibrous hyperplasia; ICU: intensive care unit; N/E: not evaluated (final outcome)

In Group 1, oral lesions had a prevalence of 5.2%, reaching 7.1% prior to the final outcome. In Group 2, the prevalence of oral mucosal changes related to hospitalisation was 9.7% in the first evaluation, increasing to 24.7% prior to the final outcome (Table 4).

**Table 4.** Oral alterations in the patients evaluated

Oral alterations – Group 1					
		First evaluation			
		Yes n(%)	No n(%)	Total n(%)	*P***^(1)^**
Until final outcome	Yes	8 (5.2)	3 (1.9)	11 (7.1)	0.250
	No	0 (0)	143 (92.9)	138 (92.9)	
TOTAL		8 (5.2)	146 (94.9)	154 (100)	
		Oral alterations – Group 2			
		Yes n(%)	No n(%)	Total n(%)	
Until final outcome	Yes	15 (9.7)	23 (15.0)	38 (24.7)	< 0.001*
	No	0	116 (75.3)	116 (75.3)	
TOTAL		15 (9.7)	139 (90.3)	154 (100)	

^(1)^McNemar’s test; *Statistical significance

We sought to associate the oral lesions found in Groups 1 and 2 with the type of oxygen support needed by the patient. We observed that all oral lesions were found in patients of Group 1 as they were using high-flow nasal catheter (*P* = 0.041), whereas patients of Group 2 had a higher prevalence of oral alterations as they were intubated (*P* < 0.001) (Table 5). Moreover, the time of hospitalisation was found to be statistically associated with the presence of oral alterations in Group 2 (*P* < 0.001), whose patients spent a mean of 17.87 ± 20.62 days hospitalised (Table 6).

**Table 5.** List of oral alterations found in relation to the type of oxygen support

Type of oxygen support	Oral alterations							
	GROUP 1				GROUP 2			
	Yes n(%)	No n(%)	Total n(%)	*P*^(1)^	Yes n(%)	No n(%)	Total n(%)	*P*^(1)^
Room atmosphere	0(0)	23 (100)	23 (100)	0.041*	0(0)	23 (100)	23 (100)	<0.001*
High-flow nasal catheter	11(11)	89(89)	100(100)		15(15)	85(85)	100(100)	
Intubation	0(0)	31(100)	31(100)		23 (74.2)	8 (25.8)	31(100)	

^(1)^Pearson’s chi-square test; *Statistical significance

**Table 6.** Association between hospitalisation time and presence of oral alterations in groups 1 and 2

Group	Presence of oral alterations	Hospitalisation time (in days)		*P **^(1)^***
		n	Mean ± SD	
1	Yes	11	11.05 ± 8.18	0.856
	No	143	12.67 ± 7.47	
2	Yes	38	17.87 ± 0.62	<0.001*
	No	116	5.03 ± 0.46	

^(1)^Student’s t-test; *Statistical significance

## Discussion

In our cohort of patients hospitalised due to COVID-19, the most frequently observed changes in the oral cavity were related to hospitalisation, namely, dryness, erythema, atrophy, cracks/fissures, oropharyngeal secretions, petechiae, spontaneous bleeding, blood clots, and traumatic ulcers [21]. These oral alterations were mainly observed in patients needing respiratory assistance with oxygen support or intubation, but the most severe changes such as spontaneous bleeding and traumatic ulcers were present in the latter case [22].

Oral health care in hospitalised patients is a critical issue, especially in those undergoing orotracheal intubation, which is related to the possibility of development of ventilator-associated pneumonia [21]. Traumatic pressure ulcers on the oral mucosa of patient in ICUs are frequent and related to biomechanical factors depending on the type of endotracheal tube being used as well as on physiological factors, such as low dosage of serum albumin, alterations in the levels of haemoglobin and haematocrit [22]. As expected, the presence of pressure ulcers depends on the duration of intubation, as the longer the period of invasive mechanical ventilation the higher the risk. Likewise, ICU setting and mechanical ventilation are risk factors for development of secondary bacterial infections [23]. In turn, such infections can result in a prolonged period of mechanical ventilation and ICU stay, and an increased mortality rate [24].

Recent evidence indicates that the oral microbiome becomes dysbiotic during COVID-19 infection and hospitalization, and can persist even after viral clearance [25; 26]. As such, further assessment of how this microbial shift relates to opportunistic infections in the oral cavity as well as secondary bacterial infections in other sites is warranted. In addition, the follow-up of the oral microbiome composition and disruption as predictors for COVID-19 disease progression is promising [27–29].

With regard to the oral mucosal lesions, three patients had pre-existing ones: pilous tongue, geographic tongue and inflammatory fibrous hyperplasia. Pseudomembranous candidiasis and angular cheilitis were observed in three other patients, whose poor oral hygiene during hospitalisation could be related to the presence of opportunistic fungi. Moreover, one of the patients diagnosed with pseudomembranous candidiasis was HIV-positive and had a marked decrease in the number of CD4 + T lymphocytes.

The majority of the ulcerative lesions observed in our cohort of patients corresponded to traumatic ulcers resulting from the mechanical pressure caused by the orotracheal tube during prolonged intubation, which was observed in five patients. Considering the relationship of these lesions with the position of the tube, along with other local manifestations, and the critical health condition of the patients, these lesions were not biopsied for further investigation. None of the 154 patients had ulcerative lesion in the first oral examination after hospitalisation.

The oral symptoms of COVID-19 at the hospitalisation were limited to dysgeusia, which was observed in 17 patients (11%). During the period of hospitalisation, only two patients developed superficial ulcerative lesions covered with fibrinopurulent exudate surrounded by an erythematous halo, resembling aphthous-like ulcers [6–13, 14. One of these patients was HIV-positive, and as previously mentioned, also presented pseudomembranous candidiasis due to a marked decrease in the count of CD4 + T lymphocytes that relapsed after one week of hospitalisation when the ulcers also appeared. Nevertheless, these ulcerative lesions in both lining and keratinised mucosa, including perioral cutaneous regions, were diagnosed as recurrent herpes simplex by laboratory and clinical examination (Figure 1) [30]. These lesions receded after systemic treatment with fluconazole and acyclovir. This patient did not need invasive mechanical ventilation during hospitalisation, recovered and was discharged after 15 days.

The oral ulcers derived from recurrent HSV-1 are mainly located in keratinized tissue, and are rarely foundin non-keratinized surfaces of immunocompetent individuals [31]. However, immunocompromised patients can present intraoral HSV-1 lesions on both keratinized and non-keratinized surfaces, and some difficulty in their management is reported, especially because HSV-1 lesions are frequently misdiagnosed with recurrent aphthous stomatitis (RAS) [32]. Cytology and PCR are reliable methods to confirm HSV-1 diagnosis but a negative result can not exclude viral infection. A biopsy is recommended in this situation [31,32].

Another patient developed aphthous-like ulcer on the tongue dorsum after four days of hospitalization, which was initially diagnosed as recurrent intraoral herpes. However, despite the more typical location for an intraoral HSV-1 lesion, the patient’s clinical presentation improved and she was discharged before the definitive diagnosis of the oral lesion was made.

A recent systematic review showed that ulcerative lesions, even the vesiculobullous ones presented by these patients, are suggestive of co-infections and immune-mediated changes [15].

The presence of co-infections by HSV-1 and cytomegalovirus (CMV), which often cause clinically indistinguishable lesions from vesiculobullous and ulcerative ones in keratinised mucosa and are reported to be associated with COVID-19, has not been investigated elsewhere [8,10–12]. A definitive diagnosis of these lesions was not possible.

Conversely, some studies investigated the presence of HSV-1 by using serology tests [6], immunohistochemistry [13], saliva PCR assay [7, 14] or lesion swab [9]. Although some cases were positive for HSV-1 [7, 14], they maintained the possibility of a direct relationship with SARS-CoV-2 infection.

As for the exclusion of the diagnosis of RAS, the majority of the studies investigated the previous history of ulcerative lesions in the oral mucosa during anamnesis [7, 9, 12, 14 and found no such pattern.

A retrospective cross-sectional study reported a high risk of COVID-19 in patients with RAS, even when they were adjusted by gender, race, age and comorbidity for COVID-19, such as respiratory diseases, endocrine diseases, obesity, diabetes mellitus, vascular diseases and smoking. Nevertheless, the absolute frequency of patients with RAS is low among those diagnosed with COVID-19 [33].

The non-specific nature of the RAS regarding its clinical manifestation and diagnosis makes it difficult to disregard this hypothesis for oral ulcers in COVID-19 patient with a positive history of RAS. SARS-CoV-2 acts through pathways such as NF-kappa B to regulate positively the expression of inflammatory cytokines, chemokines and other molecules in a feedback storm of cytokines [17,34]. This can enable the emergence of these lesions since RAS ulceration of the mucosa is a result of overexpression of chemokines and pro-inflammatory cytokines [35].

The few studies evaluating histologically the lesions possibly associated with SARS-CoV-2 [6,8,13] found changes in the epithelium (e.g. vacuolation of paranuclear keratinocytes and occasional exocytosis), lamina propria (e.g. inflammatory infiltrate of lymphocytes and neutrophils) and in small-to-medium-sized vessels (e.g. occlusive thrombosis).

The non-specific histological presentation involving vacuolation of cytoplasm and nucleus of keratinocytes in the lining epithelium, and sometimes involving nuclear pleomorphism, with the lamina propria exhibiting discrete mononuclear and polymorphonuclear inflammatory infiltrates, were observed in cases of severe oral deterioration in the specimens collected during the necropsy of the patients deceased due to COVID-19 complications. This was observed even without the presence of ulcerative lesions [5].

Another study, based on necropsies of patients deceased due to COVID-19 complications, identified the presence of SARS-CoV-2 RNA in the periodontal tissue with the same non-specific histological characteristics and absence of ulcerative lesions by associating the vacuolation of cytoplasm and nucleus of keratinocytes with viral presence. However, this was not the only condition associated with such cellular alteration, since a prolonged hospitalisation can also cause histological changes [36].

In view of the absence of ulcerative lesions at the initial evaluation of our cohort of patients hospitalised with COVID-19, including cases related to opportunistic infection, intubation and prolonged hospitalisation, it is unlikely that these oral lesions are a direct manifestation of the SARS-CoV-2 infection or a marker of COVID-19 progression. The emergence of oral lesions related to intubation and hospitalisation in this study highlights the critical importance of multidisciplinary teams providing care to COVID-19 patients both during active infection and in recovery [37]. Such approach is critical to provide support in reducing the morbidity rate, the period of hospitalization, the use of antimicrobials, and the economic impact associated with the pandemic.
